# Antioxidant, Anti-Glycation and Anti-Inflammatory Activities of Phenolic Constituents from *Cordia sinensis*

**DOI:** 10.3390/molecules161210214

**Published:** 2011-12-08

**Authors:** Nawal Al-Musayeib, Shagufta Perveen, Itrat Fatima, Muhammad Nasir, Ajaz Hussain

**Affiliations:** 1 Department of Pharmacognosy, College of Pharmacy, King Saud University, Riyadh, P.O. Box 2457, Riyadh 11451, Saudi Arabia; 2 International Centre for Chemical Sciences, H.E.J. Research Institute of Chemistry, University of Karachi, Karachi-75270, Pakistan; 3 Department of Chemistry, University of Karachi, Karachi-75270, Pakistan

**Keywords:** *Cordia sinensis*, antioxidant, anti-inflammatory, anti-glycation

## Abstract

Nine compounds have been isolated from the ethyl acetate soluble fraction of *C. sinensis*, namely protocatechuic acid (**1**), *trans*-caffeic acid (**2**), methyl rosmarinate (**3**), rosmarinic acid (**4**), kaempferide-3-*O*-*β*-D-glucopyranoside (**5**), kaempferol-3-*O*-*β*-D-glucopyranoside (**6**), quercetin-3-*O*-*β*-D-glucopyranoside (**7**), kaempferide-3-*O*-α-L-rhamnopyranosyl (1→6)-*β*-D-glucopyranoside (**8**) and kaempferol-3-*O*-α-L-rhamno-pyranosyl (1→6)-*β*-D-glucopyranoside (**9**), all reported for the first time from this species. The structures of these compounds were deduced on the basis of spectroscopic studies, including 1D and 2D NMR techniques. Compounds **1–9** were investigated for biological activity and showed significant anti-inflammatory activity in the carrageen induced rat paw edema test. The antioxidant activities of isolated compounds **1–9** were evaluated by the DPPH radical scavenging test, and compounds **1**, **2**, **4** and **7–9** exhibited marked scavenging activity compared to the standard BHA. These compounds were further studied for their anti-glycation properties and some compounds showed significant anti-glycation inhibitory activity. The purity of compounds **2–5**, **8** and **9** was confirmed by HPLC. The implications of these results for the chemotaxonomic studies of the genus *Cordia* have also been discussed.

## 1. Introduction

**Figure 1:**
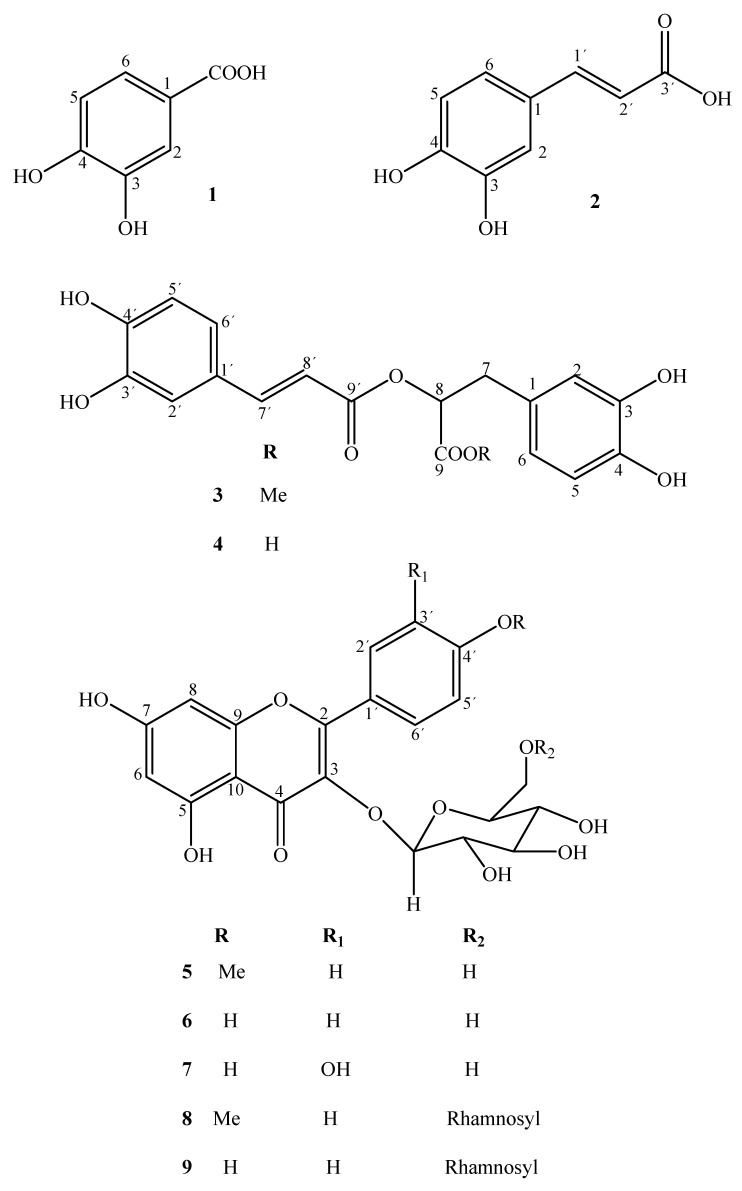
Structures of compounds **1–9**.

The genus *Cordia* belongs to the family Boraginaceae, with some 300 species distributed worldwide, mostly in the warmer regions of the World [1]. According to a literature survey, several uses in traditional medicine have been reported for different *Cordia* species [2,3,4,5,6]. The ethnopharmacological and chemotaxonomic importance of the genus *Cordia* led us to investigate the chemical constituents of one of its species, namely *Cordia sinensis*, which is a medicinal plant found widespread in the drier parts of Saudi Arabia, Africa and India [7]. The bark of *C. sinensis* is used for stomach disorders and for chest pains [8]. A literature survey revealed that very little phytochemical work has so far been carried out on *C. sinensis*. A methanolic extract of this plant showed strong toxicity in the brine shrimp lethality test and on subsequent fractionation, the major toxicity was observed in the ethyl acetate soluble sub-fraction. Further pharmacological screening of this fraction revealed potent antioxidant activity. In this paper we report the isolation of nine known compounds isolated for the first time from this plant: protocatechuic acid (**1**) [9], *trans*-caffeic acid (**2**) [10], methyl rosmarinate (**3**) [11], rosmarinic acid (**4**) [11], kaempferide-3-*O*-*β*-D-glucopyranoside (**5**) [12], kaempferol-3-*O*-*β*-D-glucopyranoside (**6**) [13], quercetin-3-*O*-*β*-D-glucopyranoside (**7**) [14], kaempferide-3-*O*-α-L-rhamnopyranosyl (1→6)-*β*-D-glucopyranoside (**8**) [13] and kaempferol-3-*O*-α-L-rhamnopyranosyl (1→6)-*β*-D-glucopyranoside (**9**) [13] (Figure 1). This study was undertaken to investigate the antioxidant potential of these compounds using the 1,1-diphenyl-2-picrylhydrazyl (DPPH) radical scavenging activity assay. *In vivo* anti-inflammatory activity for all of the compounds was also determined by the carrageen induced rat paw edema test. Finally, these compounds were studied for their anti-glycation properties. The purity of compounds **2–5**, **8** and **9** was also confirmed by analytical HPLC.

## 2. Results and Discussion

### 2.1. Compounds and Their Biological Activities

The MeOH extract of the aerial part of *C. sinensis* was divided into different sub-fractions soluble in *n*-hexane, EtOAc and *n*-BuOH. The EtOAc soluble sub-fraction was subjected to a series of column chromatography fractionations to afford compounds **1–9**. Their structures were established by MS spectrometry, UV, IR as well as NMR spectroscopy and comparison with literature data.

The antioxidant activity of compounds **1–9** was measured by the 1,1-diphenyl-2-picrylhydrazyl (DPPH) method and the results are summarized in Table 1. Compounds **1–4**, **7–9** showed significant free radical scavenging activity in this assay. Among these, rosmarinic acid (**4**), protocatechuic acid (**1**), *trans*-caffeic acid (**2**) and quercetin-3-*O*-*β*-D-glucopyranoside (**7**) were found to have high antioxidant potentials, with IC_50_ = 13.5, 14.1, 16.3 and 19.1 µM, respectively, compared to the standard BHA (IC_50_ = 44.3 µM). The antioxidant activity of flavonoids depends upon the arrangement of functional groups about the nuclear structure [15]. Accordingly, the *ortho*-dihydroxy (catechol) structure in compounds **1**, **2**, **4** plays an important role in their antioxidative function as in flavonoid **7**, and this can further explain the weak scavenging activity (IC_50_ = 53.4 µM) of kaempferol-3-*O*-*β*-D-glucopyranoside (**6**) which lacks the B-ring catechol system [16]. On the other hand, the reduced antioxidant activity of kaempferide-3-*O*-*β*-D-glucopyranoside (**5**) (IC_50_ = 55.5 µM) could be attributed to 4'-*O*-methylation that perturbs ring planarity through steric effects. The antioxidant activities of kaempferol-3-*O*-α-L-rhamnopyranosyl (1→6)-*β*-D-glucopyranoside **9** (IC_50_ = 39.5 µM) and kaempferide-3-*O*-α-L-rhamnopyranosyl (1→6)-*β*-D-glucopyranoside **8** (IC_50_ = 42.4 µM), were comparable to that of BHA [17].

**Table 1:** IC_50_ (µM) values of compounds **1–9** in the DPPH antioxidant assay.

Compounds	DPPH Scavenging Activity IC_50_^a^ [μM]
****1****	16.3 ± 0.19
****2****	14.1 ± 0.14
****3****	22.7 ± 0.17
****4****	13.5 ± 0.21
****5****	55.5 ± 0.12
****6****	53.4 ± 0.88
****7****	19.1 ± 0.90
****8****	42.4 ± 0.91
****9****	39.5 ± 0.85
****BHA**^b^**	44.3 ± 0.09

^a^ Values ± SEM (standard mean error of three assays); ^b^ Standard DPPH scavenging activity.

The oxidation process is believed to play an important role in advanced glycation as endproducts (AGEs) formation [18]. On the basis of a literature search, several natural phenolic compounds known to possess antioxidative properties, such as curcumin, rutin, garcinol and arbutin have been found to have strong antiglycation activity [19,20]. We report here for the first time the anti-glycation properties of the plant phenolics **1–9**, obtained from *C. sinensis* (Table 2).

**Table 2:** Anti-glycation activity of compounds **1–9**.

Compounds	% Inhibition
**1**	68.0
**2**	69.2
**3**	88.4
**4**	87.3
**5**	76.5
**6**	74.0
**7**	71.2
**8**	80.7
**9**	79.0
**Rutin** ^a^	86.0

^a^ Used as Standard.

Moreover, evaluation of the anti-inflammatory potential of the isolated compounds on carrageen-induced rat paw edema showed significant the potent anti-inflammatory activity of compounds **5** and **6** (62.4% and 59.6%, respectively). These flavonol glycosides were evidently more active than diclofenac sodium, which showed 57.6% inhibition of carrageen-induced rat paw edema, while compound **1** (55.0%) showed an effect comparable to that of the reference compound. The other tested compounds **2**, **7**, **8** and **9** displayed lower percentages of inhibition in the 38.4–51.2% range and were therefore less active than diclofenac sodium in this assay (Table 3) [21].

**Table 3:** Anti-inflammatory potential of compounds **1–9** in carrageen induced paw edema of rats.

Group (3 rats in each)	Treatment 100 mg/kg	Edema Volume (V_c_ = V_f_ − V_0_)	Percent Inhibition (%)
1	Cage-1 control	0	
2	Diclofenac Sodium	0.22 ± 0.05	57.6
3	**1**	0.23 ± 0.04	55.0
4	**2**	0.26 ± 0.19	50.0
5	**3**	-	-
6	**4**	-	-
7	**5**	0.24 ± 0.13	62.4
8	**6**	0.21 ± 0.11	59.6
9	**7**	0.25 ± 0.08	51.2
10	**8**	0.35 ± 0.21	43.5
11	**9**	0.32 ± 0.07	38.4

### 2.2. Chemotaxonomic Significance

The genus *Cordia* is known for the presence of a variety of secondary metabolites. Previous investigations led to the isolation of cytotoxic monoterpenes from the roots of *C. curassavica* and *C. globosa* [3,4], meroterpenoid naphthoquinones from the roots of *C. linnaei* [6], anti-inflammatory sesquiterpenes from *C. trichotoma* and *C. verbenacea* [5,22], abietane type diterpenes from *C. latifolia* [23], triterpenes from *C. spinescens, C. multispicata* and from *C. verbenacea* [24,25,26], saponins from *C. piauhiensis* [27,28,29],antifungal and larvicidal phenylpropanoid derivatives from the root bark of *C. alliodora* [2], essential oils from *C. cylindrostachya* [30], flavonoids from *C. dichotoma*, *C. obliqua* and from the flowers of *C. dentate* [31,32,33], flavonones from the aerial parts of *C. glosbosa*, *C. oblique*, *C. francisci*, *C. martinicensis*, *C. myxa* and *C. serratifolia* [34], pyrrolizidine alkaloids from *C. myxa* and glutarimide alkaloids from *C. globifera* [35,36], lignan from *C. rufescens* [37], rosmarinic acid from *C. americana* and different aromatic compounds from C. latifolia and *C. dentate* [38,39,40]. The present study reported the isolation of compounds **1–9** from the aerial parts of *C. sinensis*. This adds kaempferol type flavonoid glycosides **6–9** to the list of genus *Cordia*, while all of the other compounds were isolated for the first time from the plant *C. sinensis*. The isolation and identification of these compounds from *C. sinensis* represents a contribution to the phytochemical analysis of the components of the plant and it may be useful in further chemotaxonomic studies on the genus *Cordia*.

## 3. Experimental

### 3.1. General

The UV and IR spectra were recorded on Hitachi-UV-3200 and JASCO 320-A spectrometers, respectively. The ^1^H-, ^13^C-NMR and 2D-NMR spectra were recorded on a Bruker AMX-400 spectrometer with tetramethylsilane (TMS) as an internal standard. Chemical shifts are in given ppm (*δ*), relative to tetramethylsilane as an internal standard and scalar coupling constants (*J*) are reported in Hertz. FAB and HRFABMS (neg. ion mode, matrix: glycerol) were registered on a JEOL JMS-HX110 mass spectrometer. Thin layer chromatography (TLC) was performed on precoated silica gel F_254_ plates (E. Merck, Darmstadt, Germany); the detection was done at 254 nm and by spraying with ceric sulphate reagent. All chemicals were purchased from Sigma Chemical Company (St. Louis, MO, USA). Analytical HPLC was performed with Shimadzu HPLC equipment comprising an LC-10 ATVP pump, SPD-M.OA AVP photodiode-array (PDA) detector set at 271 nm, and a Rheodyne 7010 SIL-10 ADVP injector equipped with a 50-μL sample loop. Compounds were injected on a 250 mm × 4.0 mm, 5 μm particle size LiChrospher 100 RP-18 column from Merck (Darmstadt, Germany). The mobile phase was a 1:1 mixture of acetonitrile (ACN) and 0.05% aqueous trifluoroacetic acid at a flow rate of 1.0 mL min^−1^. Before use the mobile phase was filtered and degassed.

### 3.2. Plant Material

The whole plant of *Cordia sinensis* (Boraginaceae) was collected from Riyadh (Saudi Arabia) and identified by Dr. M. Atiqur Rahman, Plant Taxonomist, College of Pharmacy, King Saud University. A voucher specimen No.30 was deposited in the herbarium of the Department of Pharmacognosy, King Saud University, Riyadh, Saudi Arabia.

### 3.3. Extraction and Isolation

The shade dried plant material (1.5 kg) was extracted with methanol (6.0 L, thrice) at room temperature. The combined methanolic extract was evaporated under reduced pressure to give a thick gummy mass (70 g) that was suspended in water and successively extracted with *n*-hexane, ethyl acetate and *n*-butanol to afford the corresponding sub-fractions. The ethyl acetate soluble sub-fraction (15 g) was subjected to column chromatography eluting with CHCl_3_, CHCl_3_-MeOH and MeOH in increasing order of polarity to obtain five fractions I–V. Fraction I obtained from CHCl_3_-MeOH (9.9:0.1) was further purified by column chromatography eluting with CHCl_3_-MeOH (9.7:0.3) to afford compounds **1** (10 mg) and **2** (25 mg) from the top and the tail fractions, respectively. Fraction II obtained from CHCl_3_-MeOH (9.8:0.2) was a mixture of two components, which were separated by column chromatography using the solvent system CHCl_3_-MeOH (9.5:0.5) to afford compounds **3** (18 mg) and **4** (25 mg) from the top and the tail fractions, respectively. Fraction III obtained from CHCl_3_-MeOH (9.5:0.5) was further purified by Sephadex LH-20 column chromatography eluting with H_2_O-MeOH (8.0:2.0) to afford compounds **5** (19 mg) and **6** (10 mg). Fraction IV obtained from CHCl_3_-MeOH (9.3:0.7) was quite pure and rechromatography using the solvent system CHCl_3_-MeOH (9.0:1.0) gave compound **7** (11 mg). Fraction V obtained from CHCl_3_-MeOH (9.0:1.0) was further purified by Sephadex LH-20 column chromatography eluting with H_2_O-MeOH (8.5:1.5) to afford compounds **8** (20 mg) and **9** (20 mg). Compounds **1–9** were identified through comparison of their physical and spectral data with those reported in the literature. The absolute configuration of the sugar moieties in compounds **5–9** were determined by acid hydrolysis and the separated sugars were identified as L-rhamnose and D-glucose through co-TLC with authentic samples and the sign of the optical rotation [α]^25^_D_ = +7*.*8 to +7.9 for L-rhamnose and [α]^25^_D_ = +52*.*0 to +52.2 for D-glucose, respectively.

### 3.4. HPLC Analysis of the Purity of Compounds ***2**–**5***, ***8*** and ***9***

**Figure 2:**
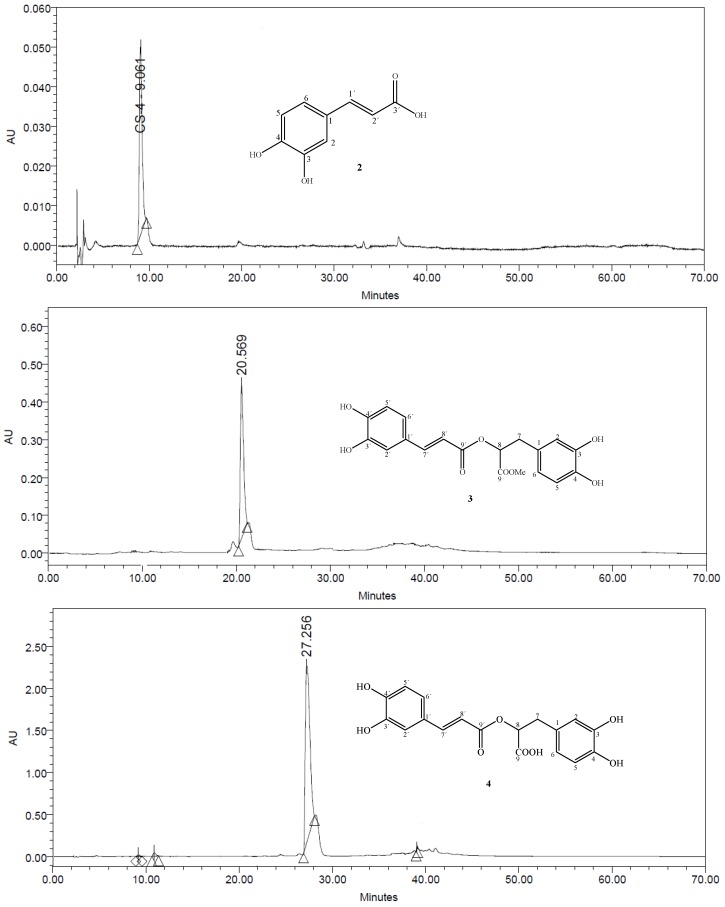

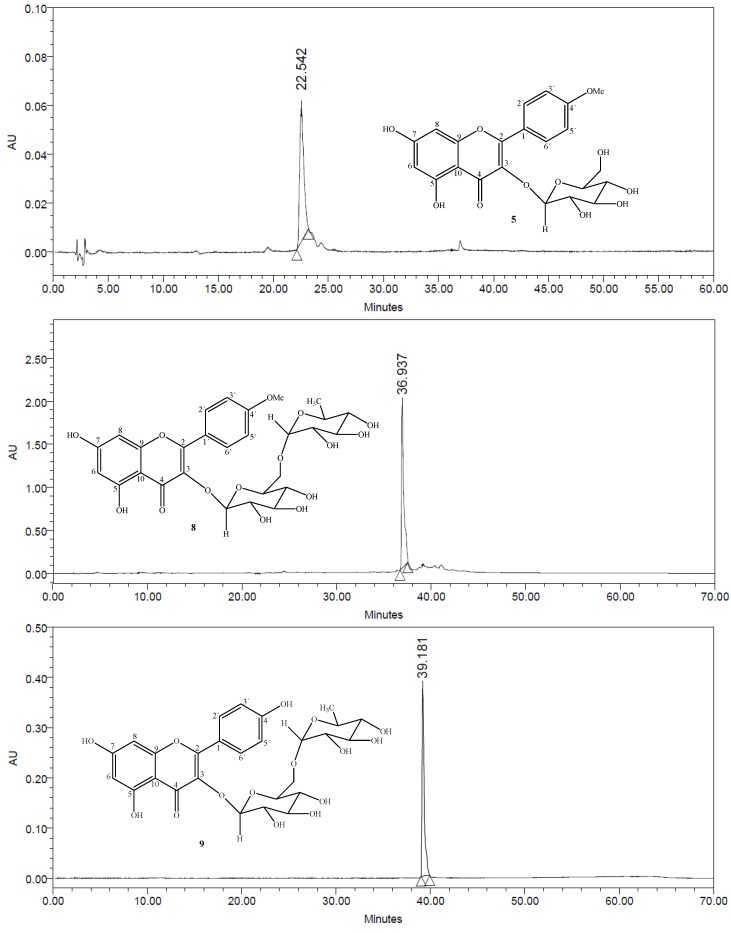
HPLC chromatograms of compounds **2–5**, **8**, **9**.

Compounds **2–5**, **8** and **9** (each 2.0 mg) was dissolved in Millipore water (HPLC grade, 5 mL) and the solution was filtered through a 0.45 μm Millipore filter. Compound **2** was detected at R*t* 9.061 min, compound **3** was detected at R*t* 20.569 min, compound **4** was detected at R*t* 27.256 min, compound **5** was detected at R*t* 22.542 min, compound **8** was detected at R*t* 36.937 min, while compound **9** appeared at R*t* 39.181 min (Figure 2). Compounds **1**, **6** and **7** were not isolated in sufficient amounts, so the purity of these compounds was not checked by HPLC.

### 3.5. Spectral Data

*Protocatechuic acid* (**1**). White crystals; m.p. 199–201 °C; HREIMS: *m/z* = 154.0258 (calc. for C_7_H_6_O_4_, 154.0266). ^1^H-NMR (CD_3_OD, 500 MHz): *δ* 6.80 (d, 1H, *J* = 8.1 Hz, 5-H), 7.30 (dd, 1H, *J* = 2.0, 8.1 Hz, 6-H), 7.46 (d, 1H, *J* = 2.0 Hz, 2-H); ^13^C-NMR (CD_3_OD, 125 MHz): *δ* 167.5 (COOH), 150.1 (C-4), 145.0 (C-3), 122.0 (C-6), 121.8 (C-1), 116.7 (C-2), 115.3 (C-5).

*trans -Caffeic acid* (**2**). White solid; m.p. 129–130 °C; HREIMS: *m/z* = 180.0391 (calc. for C_9_H_8_O_4_, 180.0423). ^1^H-NMR (CD_3_OD, 500 MHz): *δ* 7.51 (1H, d, *J* = 15.9 Hz, H-1°), 6.23 (1H, d, *J* = 15.9 Hz, H-2°), 7.14 (1H, d, *J* = 1.8 Hz, H-2), 7.02 (1H, dd, *J* = 1.8, 8.1 Hz, H-6), 6.85 (1H, d, *J* = 8.1 Hz, H-5); ^13^C-NMR (CD_3_OD, 125 MHz): *δ* 125.2 (C-1), 115.9 (C-2), 117.0 (C-5), 122.0 (C-6), 149.6 (C-4), 147.5 (C-3), 175.0 (C-1°), 123.5 (C-2°), 156.0 (C-3°).

*Methyl rosmarinate* (**3**). Yellow amorphous powder; m.p. 164–166 °C; HRFABMS: *m/z* = 375.1072 (calc. for C_19_H_19_O_8_, 375.1080). ^1^H-NMR (CD_3_OD, 500 MHz): *δ* 7.55 (1H, d, *J* = 15.5 Hz, H-7'), 7.04 (1H, d, *J* = 2.0 Hz, H- 2'), 6.95 (H, dd, *J* = 8.5, 2.0 Hz, H-6'), 6.78 (1H, d, *J* = 8.5 Hz, H-5'), 6.70 (1H, d, *J* = 2.0 Hz, H-2), 6.69 (1H, d, *J* = 8.0 Hz, H-5), 6.57 (1H, dd, *J* = 8.0, 2.0 Hz, H-6), 6.26 (1H, d, *J* = 15.5 Hz, H-8'), 5.19 (1H, dd, *J* = 7.5, 5.0 Hz, H-8), 3.70 (3H, s, OCH_3_), 3.06 (1H, dd, *J* = 14.5, 5.5 Hz, H-7a), 3.00 (1H, dd, *J* = 14.5, 5.5 Hz, H-7b); ^13^C-NMR (CD_3_OD, 125 MHz): *δ* 172.34 (C-9), 168.50 (C-9'), 150.10 (C-4'), 148.14 (C-7'), 147.03 (C-3'), 146.37 (C-3), 145.55 (C-4), 128.89 (C-1), 127.67 (C-1'), 123.38 (C-6'), 121.92 (C-6), 117.67 (C-2), 116.66 (C-5'), 116.45 (C-5), 115.34 (C-2'), 114.23 (C-8'), 74.82 (C-8), 52.82 (OCH_3_), C-38.05 (C-7).

*Rosmarinic acid* (**4**). Yellow amorphous powder; m.p. 172–174 °C; HRFABMS: *m/z* = 361.0875 (calc. for C_18_H_17_O_8_, 361.0923). ^1^H-NMR (CD_3_OD, 500 MHz): *δ* 7.51 (1H, d, *J* = 15.5 Hz, H-7'), 7.03 (1H, d, *J* = 2.0 Hz, H-2'), 6.91 (1H, dd, *J* = 8.0, 2.0 Hz, H-6'), 6.77 (1H, d, *J* = 8.0 Hz, H-5'), 6.72 (1H, d, *J* = 2.0 Hz, H-2), 6.68 (1H, d, *J* = 8.0 Hz, H-5), 6.63 (1H, dd, *J* = 8.0, 2.0 Hz, H-6), 6.27 (1H, d, *J* = 15.5 Hz, H-8'), 5.09 (1H, dd, *J* = 10.0, 3.5 Hz, H-8), 3.10 (1H, dd, *J* = 14.5, 3.5 Hz, H-7a), 2.94 (1H, dd, *J* = 14.5, 10.0 Hz, H-7b); ^13^C-NMR (CD_3_OD, 125 MHz): *δ* 177.64 (C-9), 169.24 (C-9'), 149.50 (C-4'), 146.85 (C- 3'), 146.79 (C-7'), 146.08 (C-3), 144.93 (C-4), 131.29 (C-1), 128.12 (C-1'), 123.04 (C-6'), 121.89 (C-6), 117.63 (C-2), 116.60 (C-5'), 116.34 (C-5), 115.77 (C-8'), 115.27 (C-2'), 77.79 (C-8), 38.93 (C-7).

*Kaempferide-3-O-β-D-glucopyranoside* (**5**). Yellow amorphous powder; m.p. 165–167 °C; HRFABMS: *m/z* = 461.1072 (calc. for C_22_H_21_O_11_, 461.1084). ^1^H-NMR: *δ* (DMSO-*d_6_*, 500 MHz,) 7.90 (2H, d, *J* = 8.4 Hz, H-2′, 6′), 6.87 (2H, d, *J* = 8.4 Hz, H-3′, 5′), 6.41 (1H, brs, H-8), 6.23 (1H, brs, H-6), 5.30 (1H, d, *J* = 7.5 Hz, H-1″), 3.2–4.3 (6H, sugar), 3.85 (3H, s, OCH_3_). ^13^C-NMR (DMSO-*d_6_*, 125 MHz): *δ* 156.9 (C-2), 133.5 (C-3), 177.6 (C-4), 161.4 (C-5), 98.8 (C-6), 164.6 (C-7), 94.2 (C-8), 156.7 (C-9), 103.9 (C-10), 121.5 (C-1′), 131.4 (C-2′, C-6′), 116.7 (C-3′, C-5′), 157.5 (C-4′), 101.5 (C-1″), 74.3 (C-2″), 76.5 (C-3″), 70.3 (C-4″), 76.0 (C-5″), 62.2 (C-6″), 56.7 (OCH_3_). 

*Kaempferol-3-O-β-D-glucopyranoside* (**6**). Yellow amorphous powder; m.p. 176–178 °C; HRFABMS: *m/z* = 447.0897 (calc. for C_21_H_19_O_11_, 447.0927). ^1^H-NMR (DMSO-*d_6_*, 500 MHz): *δ* 7.91 (2H, d, *J* = 8.3 Hz, H-2′, 6′), 6.86 (2H, d, *J* = 8.2 Hz, H-3′, 5′), 6.38 (1H, brs, H-8), 6.21 (1H, brs, H-6), 5.32 (1H, d, *J* = 7.5 Hz, H-1″), 3.2-4.3 (6H, sugar). ^13^C-NMR (DMSO-*d_6_*, 125 MHz): *δ* 156.8 (C-2), 133.6 (C-3), 177.8 (C-4), 161.6 (C-5), 98.9 (C-6), 164.5 (C-7), 94.0 (C-8), 156.6 (C-9), 104.2 (C-10), 121.3 (C-1′), 131.0 (C-2′, C-6′), 115.5 (C-3′, C-5′), 160.0 (C-4′), 101.4 (C-1″), 74.5 (C-2″), 76.7 (C-3″), 70.1 (C-4″), 75.9 (C-5″), 62.3 (C-6″).

*Quercetin 3-O-β-D-glucopyranoside* (**7**). Yellow amorphous powder; m.p. 229–231 °C; HRFABMS: *m/z* = 463.0758 (calc. for C_21_H_19_O_12_, 463.0876). ^1^H-NMR (DMSO-*d_6_*, 500 MHz): *δ* 6.10 (1H, d, *J* = 1.9 Hz, H-6), 6.26 (1H, d, *J* = 1.9 Hz, H-8), 6.85 (1H, d, *J* = 7.5 Hz, H-5'), 7.57 (1H, dd, *J* = 2.0, 7.5 Hz, H-6'), 7.70 (1H, d, *J* = 2.0 Hz, H-2'), 5.10 (1H, d, *J* = 7.7 Hz, H-1''), 3.30-3.80 (6H, m, H-2'', H-3'', H-4'', H-5'', H-6''); ^13^C-NMR (DMSO-*d_6_*, 125 MHz): *δ* 158.1 (C-2), 134.9 (C-3), 179.2 (C-4), 162.8 (C-5), 100.9 (C-6), 166.8 (C-7), 94.7 (C-8), 158.8 (C-9), 105.0 (C-10), 123.2 (C-1′), 116.4 (C-2′), 146.1 C-3′), 149.8 (C-4′), 117.6 (C-5′), 122.8 (C-6′) , 101.5 (C-1″), 74.5 (C-2″), 76.6 (C-3″), 70.3 (C-4″), 77.2 (C-5″), 62.1 (C-6″). 

*Kaempferide-3-O-α-L-rhamnopyranosyl (1→6)-β-D-glucopyranoside* (**8**). Yellow amorphous powder; m.p. 202–204 °C; HRFABMS: *m/z* = 607.1578 (calc. for C_28_H_31_O_15_, 607.1663). ^1^H-NMR (DMSO-*d_6_*, 500 MHz): *δ* 7.95 (2H, d, *J* = 8.5 Hz, H-2′, 6′), 6.87 (2H, d, *J* = 8.5 Hz, H-3′, 5′), 6.44 (1H, br.s, H-8), 6.25 (1H, br.s, H-6), 5.30 (1H, d, *J* = 7.4 Hz, H-1″), 4.38 (1H, br.s, H-1″′), 2.9-4.5 (10H, m, sugar), 3.82 (3H, s, OCH_3_), 1.12 (3H, d, *J* = 6.2 Hz, CH_3_). ^13^C-NMR (DMSO-*d_6_*, 125 MHz): *δ* 156.4 (C-2), 133.1 (C-3), 177.2 (C-4), 161.1 (C-5), 98.7 (C-6), 164.0 (C-7), 93.7 (C-8), 156.7 (C-9), 103.7 (C-10), 120.7 (C-1′), 131.3 (C-2′, C-6′), 116.5 (C-3′, C-5′), 158.6 (C-4′), 101.2 (C-1″), 74.0 (C-2″), 76.2 (C-3″), 69.9 (C-4″), 75.8 (C-5″), 66.7 (C-6″), 100.6 (C-1″′), 70.1 (C-2″′), 70.6 (C-3″′), 71.7 (C-4″′), 68.3 (C-5″′), 56.9 (OCH_3_), 17.5 (C-6″′).

*Kaempferol-3-O-α-L-rhamnopyranosyl (1→6)-β-D-glucopyranoside* (**9**). Yellow amorphous powder; m.p. 219–221 °C; HRFABMS: *m/z* = 593.1397 (calc. for C_27_H_29_O_15_, 593.1506). ^1^H- NMR (DMSO-*d_6_*, 500 MHz): *δ* 7.97 (2H, d, *J* = 8.6 Hz, H-2′, 6′), 6.89 (2H, d, *J* = 8.6 Hz, H-3′, 5′), 6.42 (1H, brs, H-8), 6.24 (1H, brs, H-6), 5.32 (1H, d, *J* = 7.6 Hz, H-1″), 4.39 (1H, brs, H-1″′), 3.0-4.4 (10H, m, sugar), 1.14 (3H, d, *J* = 6.5 Hz, CH_3_). ^13^C-NMR (DMSO-*d_6_*, 125 MHz): *δ* 156.9 (C-2), 133.5 (C-3), 177.0 (C-4), 161.4 (C-5), 99.2 (C-6), 164.4 (C-7), 93.9 (C-8), 156.5 (C-9), 103.4 (C-10), 121.8 (C-1′), 130.5 (C-2′, C-6′), 115.3 (C-3′, C-5′), 160.1 (C-4′), 101.5 (C-1″), 74.3 (C-2″), 76.5 (C-3″), 70.0 (C-4″), 75.4 (C-5″), 66.5 (C-6″), 100.7 (C-1″′), 70.2 (C-2″′), 70.6 (C-3″′), 71.7 (C-4″′), 68.1 (C-5″′), 17.5 (C-6″′).

## 4. Conclusions

Compounds **1–9** were isolated from the first time from the aerial parts of *C. sinensis* and showed significant biological activities. Furthermore the purity of compounds was also checked by HPLC and the data reported here should contribute to the phytochemical inventory of the species.

## References

[1] Thirupathi K., Kumar S.S., Raju V.S., Ravikumar B., Krishna D.R., Mohan G.K. (2008). A review of medicinal plants of the genus *Cordia*: Their chemistry and pharmacological uses. J. Nat. Remed..

[2] Ioset J.R., Marston A., Gupta M.P., Hostettmann K. (2000). Antifungal and larvicidal compounds from the root bark of *Cordia alliodora*. J. Nat. Prod..

[3] Menezes J.A., Lemos T., Pessoa O., Braz-Filho R., Montenegro R., Wilke D., Costa-Lotufo L., Pessoa C., Moraes M., Silveira E. (2005). A cytotoxic meroterpenoid benzoquinone from roots of *Cordia globosa*. Planta Med..

[4] Jean-Robert I., Andrew M., Mahabir P.G., Kurt H. (2000). Antifungal and larvicidal cordiaquinones from the roots of *Cordia curassavica*. Phytochemistry.

[5] Medeiros R., Passos G.F., Vitor C.E., Koepp J., Mazzuco T.L., Pianowski L.F., Campos M.M., Calixto J.B. (2007). Effect of two active compounds obtained from the essential oil of *Cordia verbenacea* on the acute inflammatory responses elicited by LPS in the rat paw. Br. J. Pharmacol..

[6] Ioset J.R., Marston A., Gupta M.P., Hostettmann K. (1998). Antifungal and larvicidal meroterpenoid naphthoquinones and a naphthoxirene from the roots of *Cordia linnaei*. Phytochemistry.

[7] Ahmed M.W.  (1990). Taxonomy and distribution of *Cordia sinensis* and *C. nevillii* (Boraginaceae), a widespread species pair in Africa and Asia. Nord. J. Bot..

[8] Richard M.M., Callistus K.P.O., Nick O.O., Paul O.O. (2010). Antitubercular and phytochemical investigation of methanol extracts of medicinal plants used by the Samburu community in Kenya. Trop. J. Pharm. Res..

[9] Buniyamin A.A., Doris N.O., Eric K.O. (2007). Isolation and characterization of two phenolics compounds from stem bark of *Musanga cecropiodes* R. brown (Moraceae). Acta Pol. Pharm..

[10] Nedime D., Seckin O., Esra U., Yasar D., Mustafa K. (2001). The Isolation of Carboxylic Acids from the Flowers of *Delphinium formosum*. Turk. J. Chem..

[11] Eun-Rhan W., Mei S.P. (2004). Antioxidative constituents from *Lycopus lucidus*. Arch. Pharm. Res..

[12] Lin L., Feng-Rui S., Rong T., Yong-Ri J., Zhi-Qiang L., Shu-Ying L. (2010). Studies on the homolytic and heterolytic cleavage of kaempferol and kaempferide glycosides using electrospray ionization tandem mass spectrometry. Rapid Commun. Mass Sp..

[13] Amal M.Y.M., Ahmed I.K., Mahmoud A.S. (2009). Isolation, structural elucidation of flavonoid constituents from *Leptadenia pyrotechnica* and evaluation of their toxicity and antitumor activity. Pharm. Biol..

[14] Choi W.H., Park W.Y., Hwang B.Y., Oh G.J., Kang S.J., Lee K.S., Ro J.S. (1998). Phenolic compounds from the stem bark of *Cornus walteri* Wagner. Kor. J. Pharmacog..

[15] Kelly E.H., Anthony R.T., Dennis J.B. (2002). Flavonoid antioxidants: Chemistry, metabolism and structure-activity relationships. J. Nutr. Biochem..

[16] Saskia A.B.E.A., Marcel J.D.G., Dirk-Jan B., Michel N.J.L.T., Gabrielle D.O.K., Wim J.F.V., Aalt B. (1996). A quantum chemical explanation of the antioxidant activity of flavonoids. Chem. Res. Toxicol..

[17] Gulcin I., Alici H.A., Cesur M. (2005). Determination of *in vitro* antioxidant and radical scavenging activities of protocol. Chem. Pharm. Bull..

[18] Rahbar S., Figarola J.L. (2003). Novel inhibitors of advanced glycation endproducts. Arch. Biochem. Biophys..

[19] Yamaguchi F., Ariga T., Yoshimura Y., Nakazawa H. (2000). Antioxidative and antiglycation activity of garcinol from *Garcinia indica* fruit rind. J. Agric. Food Chem..

[20] Kim H.Y., Kim K. (2003). Protein glycation inhibitory and antioxidative activities of some plant extracts *in vitro*. J. Agric. Food Chem..

[21] Christopher J.M. (2003). Inflammation Protocols, carrageenan-induced paw oedema in the rats and mouse. Methods Mol. Biol..

[22] Jane E.S.A.M., Telma L.G.L., Edilberto R.S., Raimundo B.F., Otília D.L.P. (2001). Trichotomol, a New Cadinenediol from *Cordia trichotoma*. J. Braz. Chem. Soc..

[23] Bina S.S., Sobiya P., Sabira B. (2006). Two new abietane diterpenes from *Cordia latifolia*. Tetrahedron.

[24] Nakamura N., Kojima S., Lim Y.A., Meselhy M.R., Hattori M., Gupta M.P., Correa M. (1997). Dammarane-type triterpenes from *Cordia spinescens*. Phytochemistry.

[25] Vincent V.V., David L., Raymond Z., Amabile K.M., Sylvio P. (1982). Cordialin A and B, two new triterpenes from *Cordia verbenacea* DC. J. Chem. Soc. Perkin Trans. 1.

[26] Kuroyanagi M., Kawahara N., Sekita S., Satake M., Hayashi T., Takase Y., Masuda K. (2003). Dammarane-type triterpenes from the Brazilian medicinal plant *Cordia multispicata*. J. Nat. Prod..

[27] Renata P.S., Edilberto R.S., Daniel E.A.U., Otilia D.L.P., Francisco A.V., Raimundo B.F. (2007). 1H and 13C NMR spectral data of new saponins from *Cordia piauhiensis*. Magn. Reson. Chem..

[28] Renata P.S., Francisco A.V., Telma L.G.L., Edilberto R.S., Raimundo B.F., Otilia D.L.P. (2003). Structure elucidation and total assignment of 1H and 13C NMR data for a new bisdesmoside saponin from *Cordia piauhiensis*. Magn. Reson. Chem..

[29] Renata P.S., Telma L.G.L., Otilia D.L.P., Raimundo B.F., Edson R.F., Francisco A.V., Edilberto R.S. (2005). Chemical constituents of *Cordia piauhiensis*—Boraginaceae. J. Braz. Chem. Soc..

[30] Fun C., Svendsen A.B.  (1990). The essential oil of *Cordia cylindrostachya* Roem. & Schult. grown on Aruba. J. Essen. Oil Res..

[31] Kuppast I.J., Nayak P.V. (2006). Wound healing activity of *Cordia dichotoma* Forst. f. fruits. Nat. Prod. Rad..

[32] Agnihotri V.K., Srivastava S.D., Srivastava S.K., Pitre S., Rusia K. (1987). Constiuents of *Cordia obliqua* as potential anti-inflammatory agents. Indian J. Pharm. Sci..

[33] Wang Y., Ohtani K., Kasai R., Yamasaki K. (1996). Flavonol glycosides and henolics from leaves of *Cordia dichotoma*. Nat. Med..

[34] Sâmia A.S.S., Maria F.A., Josean F.T., Emídio V.L.C., Jose M.B.F., Marcelo S.S.  (2010). Flavanones from aerial parts of *Cordia globosa* (Jacq.) Kunth, Boraginaceae. Rev. Bras. Farmacogn..

[35] Afzal M., Obuekwe C., Khan A.R., Barakat H. (2007). Antioxidant activity of *Cordia myxa* L. and its hepatoprotective potential. Electron. J. Environ. Agric. Food Chem..

[36] Parks J., Gyeltshen T., Prachyawarakorn V., Mahidol C., Ruchirawat S., Kittakoop P. (2010). Glutarimide alkaloids and a terpenoid benzoquinone from *Cordia globifera*. J. Nat. Prod..

[37] Samia A.S.S., Augusto L.S., Maria F.A., Emidio V.L.C., Jose M.B.F., Marcelo S.S., Raimundo B.F.  (2004). A new arylnaphthalene type lignan from *Cordia rufescens* A. DC. (Boraginaceae). ARKIVOC.

[38] Geller F., Schmidt C., Gottert M., Fronza M., Schattel V., Heinzmann B., Werz O., Flores E.M., Merfort I., Laufer S. (2010). Identification of rosmarinic acid as the major active constituent in *Cordia americana*. J. Ethnopharmacol..

[39] Ferrari F., Monache F.D., Compagnone R., Oliveri M.C. (1997). Chemical constituents of *Cordia dentata* flowers. Fitoterapia.

[40] Sabira B., Sobiya P., Bina S.S., Shazia K., Shahina F., Musarrat R. (2011). Chemical constituents of *Cordia latifolia* and their nematicidal activity. Chem. Biodiv..

